# Assessment of diffusion tensor image quality across sites and vendors using the American College of Radiology head phantom

**DOI:** 10.1120/jacmp.v17i3.5972

**Published:** 2016-05-08

**Authors:** Zhiyue J. Wang, Youngseob Seo, Evelyn Babcock, Hao Huang, Stefan Bluml, Jessica Wisnowski, Barbara Holshouser, Ashok Panigrahy, Dennis W.W. Shaw, Nolan Altman, Roderick W. McColl, Nancy K. Rollins

**Affiliations:** ^1^ Department of Radiology Children's Medical Center Dallas Dallas TX USA; ^2^ Department of Radiology University of Texas Southwestern Medical Center Dallas Dallas TX USA; ^3^ Department of Medical Physics University of Science and Technology Daejeon Republic of Korea; ^4^ Medical Metrology Center, Korea Research Institute of Standards and Science Daejeon Republic of Korea; ^5^ Advanced Imaging Research Center, University of Texas Southwestern Medical Center Dallas Dallas TX USA; ^6^ Department of Radiology Children's Hospital Los Angeles Los Angeles CA USA; ^7^ MRI Department Loma Linda University Loma Linda CA USA; ^8^ Department of Pediatric Radiology Children's Hospital of Pittsburgh Pittsburgh PA USA; ^9^ Department of Radiology Seattle Children's Hospital Seattle WA USA; ^10^ Department of Radiology Miami Children's Hospital Miami FL USA

**Keywords:** diffusion tensor imaging, quality control, multicenter, image artifacts

## Abstract

The purpose of this study was to explore the feasibility of assessing quality of diffusion tensor imaging (DTI) from multiple sites and vendors using American College of Radiology (ACR) phantom. Participating sites (Siemens (n=2), GE (n=2), and Philips (n=4)) reached consensus on parameters for DTI and used the widely available ACR phantom. Tensor data were processed at one site. $B_{0}$ and eddy current distortions were assessed using grid line displacement on phantom Slice 5; signal‐to‐noise ratio (SNR) was measured at the center and periphery of the b=0 image; fractional anisotropy (FA) and mean diffusivity (MD) were assessed using phantom Slice 7. Variations of acquisition parameters and deviations from specified sequence parameters were recorded. Nonlinear grid line distortion was higher with linear shimming and could be corrected using the 2nd order shimming. Following image registration, eddy current distortion was consistently smaller than acquisition voxel size. SNR was consistently higher in the image periphery than center by a factor of 1.3–2.0. ROI‐based FA ranged from 0.007 to 0.024. ROI‐based MD ranged from $1.90\times 10^{-3}$ to $2.33\times 10^{-3}{\text{mm}}^{2}/s(\text{median}=2.04\times 10^{-3}{\text{mm}}^{2}/s)$. Two sites had image void artifacts. The ACR phantom can be used to compare key quality measures of diffusion images acquired from multiple vendors at multiple sites.

PACS number(s): 87.57.‐s, 87.19.lf

## I. INTRODUCTION

Diffusion tensor imaging^(1)^ (DTI) is sensitive to microstructural changes that occur in cerebral white matter with normal maturation and aging. DTI is used for quantitative assessment of white matter in many disease states.^2^, ^3^, ^4^ When comparing diffusion tensor data acquired at multiple centers using the same or different vendors, biological differences in tensor metrics may in fact be related to technical factors due to different hardware platforms, software releases, and imaging parameters, as the major vendors of MR have different pulse sequences, parallel imaging techniques, and reconstruction algorithms. Moreover, receiver bandwidth, echo time, and slice thickness may differ substantially, while differences in RF head coils can result in differences in signal‐to‐noise ratio (SNR). Gradient performance and compensation of eddy currents also affect the image quality. While differences between vendors are expected, scanners of the same model and software release may also yield somewhat different results^(5)^ as evidenced by better scanner performance after routine preventative maintenance service. These technique‐related effects may both mask and mimic biological changes in white matter. There have been several reports comparing diffusion measurements in multicenter studies^5^, ^6^, ^7^, ^8^ documenting significant variability for scanners of the same model using essentially identical technique and parameters. Conducting a multicenter study using quantitative analysis of diffusion tensor data requires a uniform quality control procedure to ensure that: (i) data from different sites are not biased and can be combined for statistical analysis; and (ii) data of substandard quality are excluded. Image quality of diffusion tensor images is often determined by visual inspection of data from human subjects without the aid of phantom studies. Although valuable, visual inspection is retrospective, subjective, and observer‐dependant and may not detect subtle degradations in image quality, which might affect tensor metrics. To date, the accreditation of MR units by the American College of Radiology (ACR) is not adequate to ensure high‐quality DTI; the ACR does not mandate any quality control program for diffusion tensor imaging. The use of phantoms for multicenter DTI studies helps us understand site‐specific differences in the measured DTI metrics and detect suboptimal performance of scanners that may prompt service. One practical problem faced by investigators in a multicenter study is that most centers do not have a phantom specifically for DTI QA, and the phantoms provided by different vendors are not the same. To address the need for DTI quality assurance (QA) and the lack of a widely available DTI phantom, a QA protocol using the head phantom from ACR has been described recently. ^(9)^ We tested this QA protocol for diffusion imaging acquired at 3T scanners from different vendors at multiple sites and describe the results in this study. This study was conducted during the planning of a multicenter study of the neonatal brains.

## II. MATERIALS AND METHODS

### A. ACR head phantom

The ACR head phantom is commercially available to sites pursuing accreditation for MRI scanners by ACR. Detailed information about this phantom can be found in the ACR website (http://www.acr.org/Quality‐Safety/Accreditation/MRI).

The ACR phantom is filled with a solution of 10 mM ${\text{NiCl}}_{2}$ and 75 mM NaCl. As the liquid is isotropic, the measured fractional anisotropy (FA) value should be zero, although FA may be increased by noise. The mean diffusivity (MD) of water depends on the temperature and concentration of the electrolytes in the solution.^10^, ^11^, ^12^ For pure water at 25°C, the diffusion coefficient is $2.30\times 10^{-3}{\text{mm}}^{2}/s$,^(10)^ and increases with the temperature at a rate of $0.057\times 10^{-3}{\text{mm}}^{2}/(s\times {\;}^{\circ }C)$. Adding 75 mM of NaCl to water should decrease the water MD value by $0.01\times 10^{-3}\;{\text{mm}}^{2}/s$.^(12)^ The effect of 10 mM ${\text{NiCl}}_{2}$ on water diffusion is negligible due to its low concentration, compared to that of 75 mM NaCl.

### B. Participation sites

Eight sites in the United States participated in this study and each site is identified by a site ID. The scanners employed at each site are listed in Table 1, including two Siemens TrioTim scanners (Siemens AG, Erlangen, Germany), two General Electric HDxt units (GE Healthcare, Waukesha, WI), and four Philips Achieva units (Philips Healthcare, Andover, MA). There were no special considerations for the scanners chosen for the study other than that the participating sites were routinely acquiring diffusion tensor images in clinical or research studies. All sites followed recommended manufacturer maintenance schedules.

**Table 1:** Participating sites and scanner information.

*Site ID*	*Scanner Model and Make*	*Software Release*	*Receive Coil*
1	Siemens/TrioTim	Syngo MR B17	12 channel head coil
2	Siemens/TrioTim	Syngo MR B17	12 channel head coil
3	General Electric, Signa, HDxt	HD16.0 v01 1108.b	8HRBrain coil
4	General Electric, Signa, HDxt	15.0 M4A 0947.a	8HRBrain coil
5	Philips Healthcare, Achieva	R3.2.1	8 ch SENSE
6	Philips Healthcare, Achieva	R2.6.1	8 ch SENSE
7	Philips Healthcare, Achieva	R2.6.3	8 ch SENSE
8	Philips Healthcare, Achieva	R3.2.1	32 ch SENSE

### C. MRI QA data collection protocol

All studies were performed on 3T MRI scanners. Although it is desirable to use identical protocols for data acquisition, there are inherent differences between the scanners in both hardware and software. Participating sites reached consensus on imaging parameters for a clinical DTI study of infants which allow differences between different scanners. The key parameters for protocols specified for Siemens, GE, and Philips include $b=700\;\text{mm}/s^{2}$, 30 diffusion encoding directions, and one acquisition. The parallel imaging method is ASSET for GE, GRAPPA for Siemens, and SENSE for Philips, all with an acceleration factor of 2. The protocol allowed the GE scanners to use a larger slice thickness (2.4 mm) than Siemens and Philips scanners (2.0 mm), to accommodate the difference between scanners. Different TE is also allowed for different vendors. The site‐specific actual acquisition parameters are listed in Table 2. Autoshim was used in all studies.

Each site acquired the DTI data using the ACR head phantom. The ACR phantom was brought to the scanner room to reach thermal equilibrium for at least 24 hrs and was positioned according to ACR specifications (see “Site Scanning Instructions for Use of the MR Phantom for the ACRTM MRI Accreditation Program”, September 24 2013, http://www.acr.org/~/media/ACR/Documents/Accreditation/MRI/LargePhantomInstructions.pdf).

**Table 2:** Actual DTI acquisition parameters

*Site ID*	*Acquisition Voxel Volume (mm^3^)*	*FoV (mm)*	*Slice Thickness (mm)*	*Acquisition Matrix*	*Bandwidth (Hz/pixel)*	*TR/TE (ms)*
1	7.6	250	2.0	128×128	1563	7100/88
2	7.1	240	2.0	128×128	1563	9900/92
3	17.3	240	3.0	100×100	1953	8000/82
4	9.6	240	2.4	120×120	1953	8000/82
5	8.0	200	2.0	100×100	2742	8000/74
6	8.0	200	2.0	100×100	2742	8000/74
7^a^	8.0	200	2.0	100×100	2742	8000/74
8^a^	8.0	224	2.0	112×112	2928	8000/75

a 32 gradient encoding directions.

There were minor deviations in the actual acquisition parameters among the sites, as shown in Table 2. The table also lists the image acquisition bandwidth employed at each site, which was not specified in the protocol. The acquisition bandwidth ranged from 1563 to 2928 Hz/pixel. Six of the eight sites used specified 30 direction encoding scheme, with two sites limited to the 32 direction scheme provided by the vender. The TE value ranged from 72 to 92 ms. Site 3 used thicker slice thickness than specified by the protocol. There were also small deviations of in‐plane data acquisition resolution from the specification (Sites 1, 2, and 3).

### D. Data analysis

The DTI raw data in DICOM format was sent to the Imaging Processing Lab in our institution. Data processing was performed using an internally developed program written in IDL (IDL version 6.1, Research Systems Inc, Norwalk, CT) unless indicated otherwise. Data acquisition information contained in the DICOM header was reviewed. The DICOM data was converted into the Philips par/rec format and registered using Philips vendor proprietary software (Philips Research Imaging Development Environment (PRIDE)). The resulting images were analyzed at Slice 5 (a slice with square grid lines) and Slice 7 (a uniform slice) as defined by ACR (see “Site Scanning Instructions for Use of the MR Phantom for the ACRTM MRI Accreditation Program” in the website mentioned above) to evaluate several aspects of the data quality: (i) SNR of the b=0 image at the center and peripheries of the phantom on Slice 7;^(9)^ (ii) geometric distortion of b=0 images caused by magnetic susceptibility effects on Slice 5; (iii) geometric distortion of averaged diffusion weighted images^(9)^ relative to b=0 image on Slice 5 caused by eddy currents induced by diffusion encoding gradients; (iv) ROI‐based FA and MD measurements on Slice 7; and (v) pixel‐based FA and MD measurement on Slice 7. These analyses closely follow a previous work^(9)^ with minor modifications. First, SE images used for geometry reference were not acquired in this study. In order to assess the geometric distortions, we exploit the fact that the grid lines on Slice 5 are evenly spaced, and therefore deviation from linearity of the grid line positions in the phase encoding direction due to $B_{0}$ inhomogeneity was used to assess the geometric distortion. Linear regression was performed to fit the measured grid line position along the phase encoding direction as a function of true position in units of grid line spacing, and the standard residual error was obtained as a measure of the nonlinear distortion. Second, the distortion caused by the diffusion encoding gradient is evaluated only after image registration (a standard step in the data processing pipeline) because this is most relevant to the final image quality.

### E. Statistical analysis

One‐way ANOVA was used to assess if there were significant vendor‐specific differences in results. This test was not used for geometric distortions on the b=0 image because consistent shim setting was not available from all sites. For other measures which are not sensitive to shimming, if two values were available for one scanner (one with and one without higher order shim), the average value entered the ANOVA test.

## III. RESULTS

### A. SNR

The SNR of the b=0 images at the phantom and peripheries on Slice 7 are listed in Table 3. Since the SNR is directly proportional to the voxel size, the SNR values were also normalized by the acquisition voxel volume for comparison. All scanners showed higher median SNR at the periphery than the center. A normalized SNR was calculated for the slice center from the SNR divided by the voxel size. In one case where two acquisitions had been incidentally averaged, the SNR is also normalized by the square root of the number of acquisitions to obtain the SNR corresponding to one acquisition. This normalization did not include the effects of acquisition bandwidth on SNR. The SNR performances were comparable after normalization, ranging from 4.1 to 8.0 at the phantom center. ANOVA did not detect significant differences among different vendors (p=0.629). At the peripheries, the median value of normalized SNR of the images from each site ranged from 8.7 to 11.6. Again, ANOVA did not detect significant differences among different vendors (p=0.12) using the median peripheral SNR from each site.

**Table 3:** Measured SNR on b=0 image at Slice 7.

*Site ID*	*SNR at Slice Center*	*SNR at Slice Peripheral (median, range)*	*Normalized SNR at Slice Center*	*Normalized SNR at Slice Peripheral (median, range)*
1^b^	52	79 (53, 88)	6.8	10.4 (7.0, 11.6)
2^b^	38	62 (56, 72)	5.4	8.7 (7.9, 10.1)
3^a^	140	219 (212, 274)	5.7	9.0 (8.7, 11.2)
4^c^	72	95 (82, 110)	7.5	9.9 (8.5, 11.5)
	77	102 (71, 121)	8.0	10.6 (7.4, 12.6)
5^d^	48	89 (54, 97)	6.0	11.1 (6.8, 12.1)
	47	84 (69, 94)	5.9	10.5 (8.6, 11.8)
6^b^	33	84 (57, 103)	4.1	10.5 (7.1, 12.9)
7^d^	46	91 (74, 105)	5.8	11.4 (9.3, 13.1)
	53	83 (77, 115)	6.6	10.4 (9.6, 14.4)
8^b^	53	93 (78, 112)	6.6	11.6 (9.8, 14.0)

a Two acquisitions were averaged.

b Higher order shim was used.

c Two measurements were done in the same session; the first one used the first order shim and the second one used higher order shim.

d Two measurements were done in different days; the first one used the first order shim and the second one used higher order shim.

### B. Image distortions

**Figure 1:**
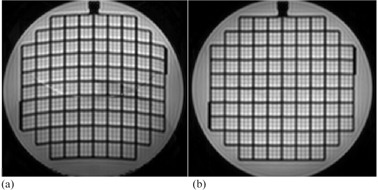
Comparison of b=0 image acquired with linear shim (a) and higher order shim (b) (images from Site 7). The use of higher order shim drastically reduced nonlinear image distortions (This figure does not display the full FoV).

All tensor data from Siemens scanners were acquired with 2nd order shim, which was the default setting. For Philips and GE sites, tensor data was initially acquired without 2nd order shim and image distortions were obvious. The size of distortion was drastically decreased with the addition of 2nd order shim (Fig. 1).

The results of nonlinear image distortion of the b=0 image at Slice 5 are listed in column 2 of Table 4. With the use of 1st order shim, the standard nonlinearity error ranged from 2.9– 6.4 mm (median=3.5 mm). With higher order shim, the range of nonlinearity error decreased to 0.4–0.8 mm (median=0.6 mm) for all scanners. ANOVA analysis was not applied to this measurement because 1) Site 3 did not provide higher order shim data, and 2) with higher order shim this distortion is very small.

The results of image distortion on diffusion weighted images caused by diffusion encoding (i.e., distortions of b=700 images relative to the b=0 image at Slice 5) are reported in columns 3 and 4 of Table 4. The median value of “mean of grid line shift” was small, with the absolute value below 0.1 mm in all cases, even though ANOVA detected vendor‐specific differences (p=0.045). There were no vender‐specific differences in the maximum value for “mean of grid line shift” (p=0.460), median of “standard deviation (SD) of grid line shift” (p=0.460), and maximum “SD of grid line shift” (p=0.510), although the worst values were all found in Site 2 in Table 4, The mean and SD of grid line shift were substantially smaller than the acquisition spatial resolution of 2 mm.

**Table 4:** Image distortions.

	*Nonlinear Image Distortion in EPI Along Phase Encoding Direction (standard error in mm)*	*Distortion by Diffusion Encoding Gradient*	
*Site ID*		*Mean of Grid Line Shift (median and range in mm)*	*SD of Grid Line Shift (median and range in mm)*
1^a^	0.7	−0.04(−0.07,0.09)	0.16 (0.01, 0.34)
2^a^	0.8	−0.04(−0.21,0.88)	0.53 (0.28, 0.93)
3	6.4	0.01(−0.13,0.25)	0.11 (0.01, 0.60)
4^b^	3.8	−0.07(−0.14,0.06)	0.24 (0.18, 0.57)
	0.6	0.01(−0.07,0.13)	0.30 (0.05, 0.57)
5^c^	3.2	0.03(−0.12,0.60)	0.17 (0.02, 0.58)
	0.6	0.00(−0.10,0.10)	0.14 (0.02, 0.34)
6^a^	0.6	−0.01(−0.16,0.10)	0.28 (0.07, 0.50)
7^c^	2.9	0.03(−0.10,0.19)	0.20 (0.03, 0.62)
	0.4	0.00(−0.12,0.07)	0.18 (0.04, 0.45)
8^a^	0.7	0.01(−0.05,0.04)	0.17 (0.09, 0.27)

a Higher order shim was used.

b Two measurements were done in the same session; the first one used the first order shim and the second one used higher order shim.

c Two measurements were done in different days; the first one used the first order shim and the second one used higher order shim.

### C. Fractional anisotropy and mean diffusivity

ROI‐based FA and MD and pixel‐based FA and MD results are listed in Table 5. The ROI‐based MD ranged from $1.90\times 10^{-3}\;\text{to}\;2.33\times 10^{-3}\;{\text{mm}}^{2}/s(\text{median}=2.04\times 10^{-3}\;{\text{mm}}^{2}/s)$. Voxel‐based MD ranged from $1.91\times 10^{-3}\;\text{to}\;2.33\times 10^{-3}\;{\text{mm}}^{2}/s(\text{median}=2.05\times 10^{-3}\;{\text{mm}}^{2}/s)$. The variability of MD most likely reflects difference in the scanner room temperatures although the possibility of miscalibration of the diffusion encoding gradient (i.e., the actual b‐value is different from the nominal b‐value) could not be ruled out. This range of MD corresponds to a temperature range from 20.7° to 25.3°C $\left(\text{median}=22.2^{\circ }C\right)$. There were no vendor specific differences in ROI‐ and voxel‐based MD values (p=0.091 and 0.110, respectively).

ROI‐based FA ranged from 0.0065 to 0.0240 (median=0.0136). Voxel‐based mean FA ranged from 0.040 to 0.067 (median=0.052). ANOVA detected vendor‐specific differences in ROI‐based FA value (p=0.036). Table 5 showed that Philips scanners consistently have higher ROI‐based FA values. Differences in pixel‐based FA value was not detected (p=0.081).

**Table 5:** FA and MD evaluated from Slice 7.

	*Fractional Anisotropy*		*Mean Diffusivity* $\left(10^{-3}\;{\text{mm}}^{2}/\text{sec}\right)$	
*Site*	*ROI‐based*	*Voxel‐based*	*ROI‐based*	*Voxel‐based*
1^a^	0.0098	0.059±0.058	1.93	1.95±0.20
2^a^	0.0136	0.067±0.040	2.00	2.02±0.09
3	0.0065	0.040±0.018	2.10	2.11±0.05
4^b^	0.0097	0.052±0.020	2.33	2.33±0.06
	0.0109	0.054±0.018	2.32	2.32±0.07
5^c^	0.0190	0.060±0.035	2.04	2.05±0.10
	0.0091	0.042±0.014	1.97	1.97±0.04
6^a^	0.0191	0.053±0.020	2.14	2.14±0.05
7^c^	0.0240	0.051±0.025	1.90	1.91±0.07
0.0210	0.046±0.019	1.96	1.96±0.04
8^a^	0.0171	0.040±0.014	2.15	2.15±0.03

a Higher order shim was used.

b Two measurements were done in the same session; the first one used the first order shim and the second one used higher order shim.

c Two measurements were done in different days; the first one used the first order shim and the second one used higher order shim.

### D. Imaging artifacts

**Figure 2:**
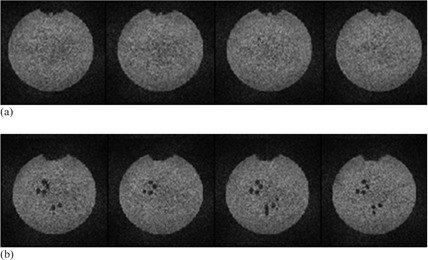
Signal void artifacts on diffusion weighted images (Site 2). Both (a) and (b) show the same four consecutive slices (approximately at Slice 7) for two different diffusion encoding directions. Signal void artifacts are prominent in (b). They appear to reflect the spoke patterns in Slices 8–11 of the ACR phantom.

We have observed signal void artifacts on diffusion‐weighted images acquired on Siemens scanners (Fig. 2). These artifacts were absent on the b=0 images, and the pattern of the signal voids varied for different diffusion encoding directions. Further analysis using the color encoded FA map reveals that the signal void region is displayed as red, indicating signal loss in the left–right diffusion encoding direction. It is possible this artifact was related to mechanical vibrations of the table during the scan which can occur in some scanners of this model when the patient weight is low, such as in pediatric or phantom studies. A retrofit kit has been developed by the manufacturer to address this issue (Siemens Medical Systems, Erlangen, Germany). The signal void artifact also appeared on some diffusion‐weighted images on slice 5 for Site 2.

At the affected areas on Slice 5, our procedure for quantifying the image distortion caused by diffusion encoding gradient could not be carried out. We excluded two regions with artifacts from this analysis for Site 2. The regions of signal loss were excluded from regions used for SNR evaluation; thus, reported SNR values were not affected. Efforts were not made to exclude artifacts regions from the FA and MD analysis.

## IV. DISCUSSION

Without careful quality control, the benefit of conducting a multicenter study may be compromised. Variability of measurements resulting from technical factors across sites may necessitate a larger sample size, thereby increasing the costs and duration of time needed to achieve statistical power in a study. When there are a limited number of participating centers contributing a large number of subjects, the potential technical variability among the centers may be controlled by using the site ID as a covariate in the statistical analysis,^(13)^ though this will still potentially increase the number of subjects needed. With many centers contributing relatively few patients, variability between sites must be minimized. There have been several reports comparing diffusion measurements in multicenter studies^5^, ^6^, ^7^, ^8^ documenting significant variability for scanners of the same model using essentially identical technique and parameters. Many aspects of scanner performance have been reported to affect the measurement results and introduce discrepancies in the measurement.^(14)^ Factors affecting scanner performance include, but are not limited to, SNR,^15^, ^16^, ^17^ susceptibility effects due to poor shimming,^(18)^ and image distortion resulting from eddy currents.^(19)^ In this study, we compared some specific products of three commercial MR vendors. The participating centers in this study had variable physics support and expertise in DTI. Therefore, we aimed for a simple study design for data acquisition and used the widely available ACR head MRI phantom.

The results of these phantom tests may be used to provide feedback to guide the sites to take corrective actions regarding protocol changes or artifact reduction for problems which may not have been as obvious during routine clinical acquisitions. As discussed previously, it is important to establish what one considers satisfactory results for QA test values.^(9)^ At this stage, more phantom data and studies correlating image quality and phantom results are needed in order to establish corrective action criteria for this QA procedure. The corrective action criteria need to be based on practical considerations and also depend on the specific aims of the study or the examination. Qualitatively speaking, the distortion caused by diffusion encoding gradient should be much smaller than the sampling voxel size. In the present DTI technology, the acquisition voxel size is mainly limited by the achievable SNR, although our results show that geometric distortion caused by eddy currents could be a limiting factor, as well. Typically the voxel‐based FA value is on the order of a few percent, which is mostly determined by the noise level. Because voxel‐based FA is unable to reveal small systematic error in FA quantification, we also carried out an ROI‐based FA measurement and surprisingly observed significant differences between vendors. In our view, the ROI‐based FA measured on the phantom should be preferably about 0.01 or lower, as have been achieved by the majority vendors in this study. In this regard, action from the MRI vendor is needed. There can be implications of measured increase in ROI‐based FA. In this study we found ROI‐based FA deviated more from zero for Philips scanners than that from other vendors (Table 5). A simple calculation showed that a plus or minus 3.5% miscalibration of diffusion encoding gradient along one direction is required to cause the measured FA to deviate from 0 to 0.02. For a fiber tract along this direction with a true FA equal to 0.5, the measured FA will be 0.482 if the gradient is too low, or 0.516 if the gradient is too high. The discrepancy between the two extremes is 3.4%. Likewise, errors in ROI‐based MD revealed in the QA test will directly affect quantification in human studies. It is important to include this assessment if quantification of individual diffusivities is a specific aim of the human study.

Many types of phantoms have been described for DTI QA purposes, including isotropic solution bottle phantom using water^(20)^ or other liquids with diffusivities similar to human brain tissues,^(21)^ and anisotropic phantoms.^22^, ^23^, ^24^, ^25^, ^26^ One primary task of DTI QA is to ensure the measured diffusivity and FA values are accurate. Anisotropic phantoms conveniently allow direct quality control of the measured non‐zero FA value and diffusion tensor orientation. Using an anisotropic phantom, a discrepancy between a nominal and actual gradient encoding direction caused by pulse programming error may be readily detected. If the encoding gradient direction is programmed correctly, same information can be extracted from data acquired with an isotropic phantom. In other words, if the measured FA value from an anisotropic phantom is found to deviate from the true value, the same measurement on an isotropic phantom should also return non‐zero values, and vice versa. Another important task of DTI QA is to ensure minimal geometric distortion, including distortions caused by diffusion sensitizing gradients, which can be identified from their dependence on the diffusion encoding orientation. For this purpose phantoms with internal structures are needed. The ACR phantom is not designed for DTI QA, especially with its solution diffusivity substantially different from normal brain gray and white matters. However, this phantom is widely and readily available, and is a better alternative to routine MRI phantoms provided by vendors. Comparing DTI QA results obtained on different vendor‐specific phantoms would be very difficult. Furthermore, the ACR phantom has internal structures that allow assessment of geometric distortions, such as was done in this study.

There are limitations in this study. Repeatability and reproducibility data were not collected from sites participating in this study. Therefore, it is difficult to assess whether the variability of all the metrics is due to the systematic difference of machines from different vendors, as mentioned in the studies by Volimar et al., Zhu et al., Fox et al., and Teipel et al.,^5^, ^6^, ^7^, ^8^ or due to random errors in the MRI measurements. Since the goal of this study was to evaluate a QC methodology for multicenter trial, knowing under what circumstance the site needs to undergo hardware/software adjustment is important. The proposed method can be used as a tool to detect system performance degradation over the course of the trial. Therefore, knowing the normal range of the parameters plays a key role, and this requires repeatability studies. Another limitation of the study is that there is no correlation between the QC results and the routine clinical DTI images, although this is not a trivial task. Consequently, it's difficult to evaluate the clinical image quality based on the QC phantom images on a given system.

This multisite investigation, although limited in the number of sites for each MR vendor, suggests that using the ACR phantom for assessment of nonlinear image distortion, eddy current distortion, and deviation from expected measurements of FA and MD is technically feasible and readily implemented. This simple QC assessment can be considered for multicenter studies entailing acquisition of diffusion images, and results can be used to trigger maintenance of the MR scanner outside the preventive maintenance schedule. For example, Site 2 may ask the service engineers to improve eddy current compensation in order to decrease the observed large distortion in Table 4.

## V. CONCLUSIONS

In the absence of an affordable widely available anisotropic phantom, the ACR phantom can be used as an alternative to assess image qualities across multiple sites. Certain patterns of characteristics can be identified for scanners from different vendors. Attention should be made to factors, including higher order shimming, in order to minimize differences between platforms. Overall, the performance of the scanners as evaluated by the phantom tests appears comparable, even though the acquisition parameters were not identical.

## ACKNOWLEDGMENTS

YS was supported by the National Research Foundation of Korea (NRF‐2014R1A1A2054037).

## COPYRIGHT

This work is licensed under a Creative Commons Attribution 4.0 International License.
